# Four new species of hangingflies (Insecta, Mecoptera, Bittacidae) from the Middle Jurassic of northeastern China

**DOI:** 10.3897/zookeys.466.8047

**Published:** 2014-12-19

**Authors:** Sulin Liu, Chungkun Shih, Dong Ren

**Affiliations:** 1 College of Life Sciences, Capital Normal University, 105 Xisanhuanbeilu, Haidian District, Beijing 100048, China

**Keywords:** *Mongolbittacus*, *Exilibittacus*, Jiulongshan Formation, Daohugou, Insect fossil

## Abstract

Two new species of *Mongolbittacus* Petrulevičius, Huang & Ren, 2007, *Mongolbittacus speciosus* **sp. n.** and *Mongolbittacus oligophlebius* **sp. n.**, and two new species of *Exilibittacus* Yang, Ren & Shih, 2012, *Exilibittacus foliaceus* **sp. n.** and *Exilibittacus plagioneurus* **sp. n.**, in the family Bittacidae, are described and illustrated based on five well-preserved fossil specimens. These specimens were collected from the late Middle Jurassic Jiulongshan Formation of Daohugou, Inner Mongolia, China. These new findings enhance our understanding of the morphological characters of early hangingflies and highlight the diversity of bittacids in the Mid Mesozoic ecosystems.

## Introduction

Bittacidae, a large family of Mecoptera commonly called hangingflies, live mainly in the temperate or warm tropical climates. The fifth tarsomere of bittacids can be folded against the fourth with the only one claw at pretarsus (Petrulevičius et al. 2007). Since this special tarsi structure is shared by a sister group of Cimbrophlebiidae (Archibald 2009; Yang et al. 2012a), it is suggested that this morphological character may be an apomorphy. So far, there are 16 extant genera with about 270 described extant species (Krzemiński 2007; Yang et al. 2012b). For fossil records, there are 28 genera comprising 52 species as summarized by Li and Ren (2009b). Since then, two genera, *Decoribittacus* Li & Ren, 2009 and *Exilibittacus* Yang, Ren & Shih, 2012, with three species have been described (Li et al. 2009a, Yang et al. 2012b). In addition, up to date, about 20 genera have been described from the Jurassic (Handlirsch 1906, 1939; Ansorge 1993; Tillyard 1933; Ren 1993, 1997; Novokshonov 1993a, 1993b, 1997; Petrulevičius et al. 2007; Li et al. 2008, 2009a; Yang et al. 2012a, 2012b). The age distribution for these fossil genera suggests that the broadest diversity of Bittacidae occurred during the Jurassic, and the earliest fossil record of Bittacidae is *Archebittacus exilis* Riek, 1955 from the Upper Triassic of Mt. Crosby, Australia (Riek 1955).

Until now, 11 fossil genera of Bittacidae from the late Middle Jurassic to the Early Cretaceous have been recorded in China: *Liaobittacus* Ren, 1993 from the Haifanggou Formation; *Megabittacus* Ren, 1997 and *Sibirobittacus* Sukatcheva, 1990 from the Yixian Formation; *Neorthophlebia* Handlirsch, 1906 from the Tuodian Formation; *Preanabittacus* Novokshonov, 1993, *Mongolbittacus* Petrulevičius, Huang & Ren, 2007, *Formosibittacus* Li, Ren & Shih, 2008, *Jurahylobittacus* Li, Ren & Shih, 2008, *Decoribittacus* Li & Ren, 2009, *Karattacus* Novokshonov, 1997, and *Exilibittacus* Yang, Ren & Shih, 2012, all from the Jiulongshan Formation. A list of 14 species in 11 genera is summarized in Table 1.

**Table 1.** A list of Bittacidae fossils described from China.

Genus	Species	Locality	Horizon/Age
*Megabittacus* Ren, 1997	*Megabittacus beipiaoensis* Ren, 1997	Beipiao, Liaoning	Yixian Fm.,K1
	*Megabittacus colosseus* Ren, 1997	Beipiao, Liaoning	Yixian Fm.,K1
	*Megabittacus spatiosus* Yang, Shih & Ren, 2012	Beipiao, Liaoning	Yixian Fm.,K1
*Sibirobittacus* Novokshonov, 1993	*Sibirobittacus atalus* Ren, 1997	Beipiao, Liaoning	Yixian Fm., K1
*Neorthophlebia* Handlirsch, 1906	*Neorthophlebia yunnanensis* Zhang & Hong, 2003	Tuodian, Yunnan	Tuodian Fm., J3
*Decoribittacus* Li & Ren, 2009	*Decoribittacus euneurus* Li & Ren, 2009	Ningcheng, Inner Mongolia	Jiulongshan Fm., J2
	*Decoribittacus stictus* Li & Ren, 2009	Ningcheng, Inner Mongolia	Jiulongshan Fm., J2
*Exilibittacus* Yang, Shih & Ren, 2012	*Exilibittacus lii* Yang, Shih & Ren, 2012	Ningcheng, Inner Mongolia	Jiulongshan Fm., J2
	*Exilibittacus plagioneurus* sp. n.	Ningcheng, Inner Mongolia	Jiulongshan Fm., J2
	*Exilibittacus foliaceus* sp. n.	Ningcheng, Inner Mongolia	Jiulongshan Fm., J2
*Formosibittacus* Li, Ren & Shih, 2008	*Formosibittacus macularis* Li, Ren & Shih, 2008	Ningcheng, Inner Mongolia	Jiulongshan Fm., J2
*Jurahylobittacus* Li, Ren & Shih, 2008	*Jurahylobittacus astictus* Li, Ren & Shih, 2008	Ningcheng, Inner Mongolia	Jiulongshan Fm., J2
*Karattacus* Novokshonov, 1997	*Karattacus longialatus* Li & Ren, 2009	Ningcheng, Inner Mongolia	Jiulongshan Fm., J2
*Liaobittacus* Ren, 1993	*Liaobittacus longantennatus* Ren, 1993	Beipiao, Liaoning	Haifanggou Fm., J2
*Preanabittacus* Novokshonov, 1993	*Preanabittacus validus* Yang, Shih & Ren, 2012	Ningcheng, Inner Mongolia	Jiulongshan Fm., J2
*Mongolbittacus* Petrulevičius, Huang & Ren, 2007	*Mongolbittacus daohugoensis* Petrulevičius, Huang & Ren, 2007	Ningcheng, Inner Mongolia	Jiulongshan Fm., J2
	*Mongolbittacus speciosus* sp. n.	Ningcheng, Inner Mongolia	Jiulongshan Fm., J2
	*Mongolbittacus oligophlebius* sp. n.	Ningcheng, Inner Mongolia	Jiulongshan Fm., J2

Herein we describe four new species of Bittacidae, based on five recently collected fossil specimens from the Jiulongshan Formation of Daohugou, Ningcheng County, Inner Mongolia, China. The section at Daohugou Village is composed of grey tuffaceous sandstone and sandy mudstone (Ren et al. 2002). This formation has yielded abundant and diverse insect fossils (Ren et al. 2010), such as Lepidoptera (Zhang et al. 2013), Mecoptera (Ren et al. 2009; Wang et al. 2012; Wang et al. 2014), Hymenoptera (Shih et al. 2010; Li et al. 2013; Wang et al. 2014), Diptera (Liu et al. 2012), Neuroptera (Wang et al. 2010) and many others insects (Gao et al. 2012).

## Material and methods

The fossil specimens were examined with a Leica M165C dissecting microscope and illustrated with the aid of a camera lucida attached to the microscope; drawings were scanned into a computer by EPSON5100 and were edited with Adobe Photoshop® CS3. Photographs of the specimens and magnified images of the details were taken with a digital camera system attached to the Leica M165C. Specimens were at times treated with ethanol (95%) on the surface to enhance the clarity and contrast. All type specimens are deposited in the Key Lab of Insect Evolution and Environmental Changes, the College of Life Sciences, Capital Normal University, Beijing, China (CNUB, Ren Dong, Curator). The wing venation nomenclature follows Byers (1979). The term of ‘bittacid cross’ is defined as the crossveins of [R_4+5_-M_1+2_, M_1+2_-M_3_] (Bechly and Schweigert 2000).

## Systematic paleontology

### Order Mecoptera Packard, 1886 Infraorder Raptipeda Willmann, 1977 Family Bittacidae Handlirsch, 1906

#### Genus Mongolbittacus

Taxon classificationAnimaliaMecopteraBittacidae

Petrulevičius, Huang & Ren, 2007

##### Type species.

*Mongolbittacus daohugoensis* Petrulevičius, Huang & Ren, 2007

##### Included species.

Type species, *Mongolbittacus speciosus* sp. n., and *Mongolbittacus oligophlebius* sp. n.

#### Mongolbittacus speciosus sp. n.

Taxon classificationAnimaliaMecopteraBittacidae

http://zoobank.org/442F8176-318C-4671-8BA1-83B449A8F4C7

Figs 1 , 2 , 3 , 4

##### Etymology.

The specific epithet is derived from a Latin word of *speciosus* (showy), highlighting the well-preserved wings in the holotype.

##### Holotype.

A male specimen well-preserved, CNU-MEC-NN2013008 P/C, part and counterpart. Body 8.8 mm long; forewing 11.3 mm long with a maximal width of 3.0 mm; hind wing 9.1 mm long with a maximal width of 3.0 mm.

##### Horizon and locality.

Jiulongshan Formation, late Middle Jurassic, Daohugou Village, Shantou Township, Ningcheng County, Inner Mongolia, China.

##### Diagnosis.

In forewing, Sc reaching the anterior margin proximad of the forking of Rs; one crossvein between C and R_1_; 1A and 2A fusing at base; and 2A sharply curving to the posterior margin.

##### Description.

**Figure 1.**
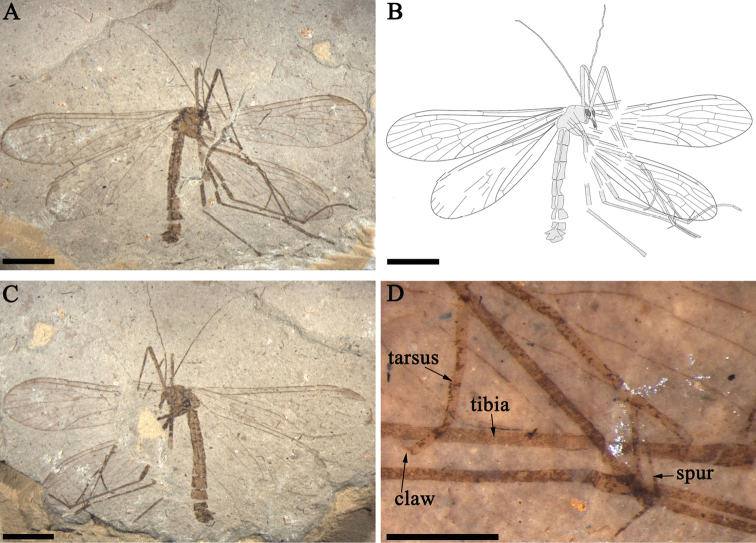
*Mongolbittacus speciosus* sp. n., holotype, CNU-MEC-NN2013008 P/C. **A** photo of part **B** line drawing of part **C** photo of counterpart **D** photo of legs, under alcohol. Scale bars represent 3 mm in **A–C**, 0.5 mm in **D.**

A male specimen in lateral view. The head oviform with robust and slender chewing mouthparts. Compound eyes large and oval. Antennae almost complete, filiform, about 6.9 mm long, comprising about twenty antennomeres; the lengths of basal antennomeres almost the same, but several apical antennomeres shorter than the basal ones. Thorax divided into pronotum, mesonotum and metanotum from the lateral view (Figs 1A–C, 4B, F).

Legs. Long and slender in lateral view, densely covered with short setae. But all legs fragmented due to poor preservation. Mesocoxa, metacoxa, trochanter visible in lateral view. Mid tibia 4.4 mm; tibial spurs long and sharp. Tarsus with 5 tarsomeres and a single pretarsal claw, but the fifth tarsomere not folded against the fourth as preserved. In addition, the second and third tarsomeres covered with a few small spines (Fig. 1A–D).

**Figure 2.**
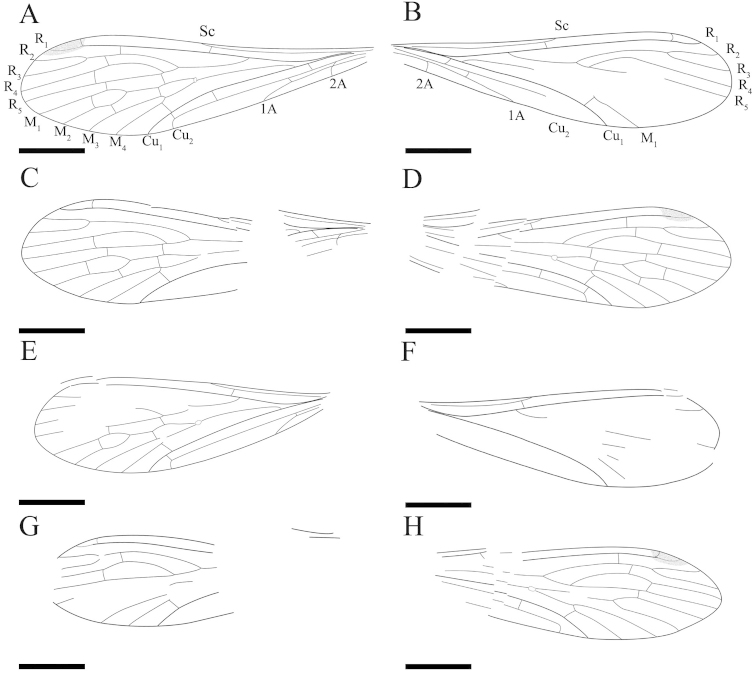
*Mongolbittacus speciosus* sp. n., holotype, CNU-MEC-NN2013008 P/C. Line drawings of part. **A** left forewing **D** right forewing **E** left hind wing **H** right hind wing. Line drawings of counterpart **B** right forewing **C** left forewing **F** right hind wing **G** left hind wing. Scale bars represent 1 mm in **A–H.**

**Figure 3.**
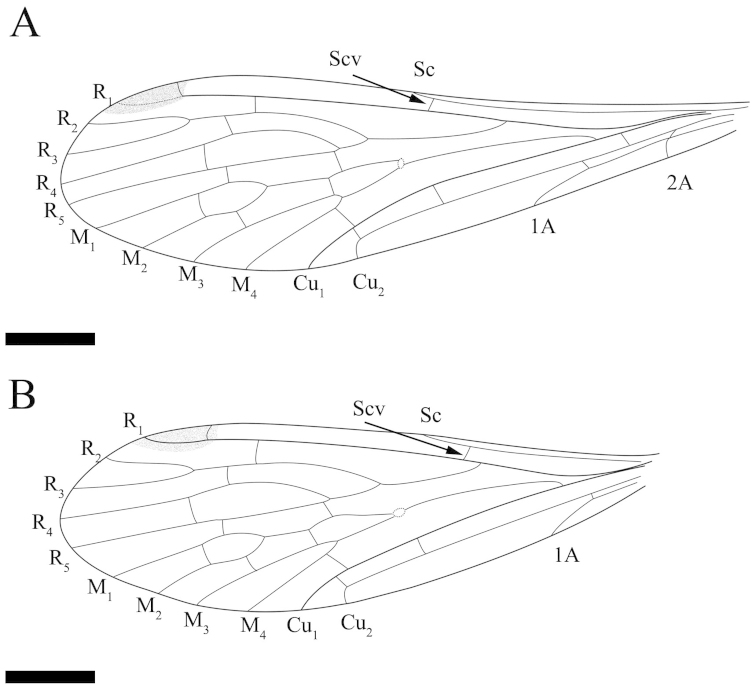
*Mongolbittacus speciosus* sp. n., holotype. **A** line drawing of forewing, composite of right and left forewings of part and counterpart **B** line drawing of hind wing, composite of right and left hind wings of part and counterpart. Scale bars represent 1 mm in **A–B.**

Forewing. No maculation, base of wing narrow. Sc short, one oblique subcostal crossvein (Scv) between Sc and R_1_; one crossvein between R_1_ and C; R_1_ smooth and reaching the dark pterostigmal area; Rs originating from R_1_ at an acute angle; one crossvein between R_1_ and R_2+3_, one crossvein between R_2+3_ and R_4_ and one crossvein between R_4_ and R_5_; the ‘bittacid cross’ not aligned, Z-shaped (in side view), and posterior part of ‘the ‘bittacid cross’ distad of the forking of M_3+4_; M with four branches and bifurcating proximad of the forking of Rs; one crossvein between R_5_ and M_1_, one between M_1_ and M_2_ and one between M_2_ and M_3_; M_4_ simple, one long and oblique crossvein between M_4_ and Cu_1_; Cu_1_ and M overlapping at base for a short distance; Cu_2_ curving sharply with a 90° angle, reaching the posterior margin; Cu_1_ and Cu_2_ almost parallel, with three crossveins between them, the first oblique crossvein located at the base of the wing, the second at the level of Scv, and the third near the sharp bending of Cu_2_. Veins 1A and 2A fusing at base, 1A reaching the posterior margin proximad of the origination of Rs from R_1_; two crossveins between 1A and Cu_2_ (Figs 2A–D, 3A).

Hind wing. Sc short, reaching the anterior margin proximad of the forking of Rs; one crossvein between R_1_ and C; One subcostal crossvein (Scv) between Sc and R_1_, one crossvein between R_1_ and R_2+3_, and one short crossvein between R_2+3_ and R_4_; R_4_ sharply bending upwards, then parallel with R_5_, one crossvein between them; the ‘bittacid cross’ not aligned, Z-shaped; M forking proximad of the bifurcation of Rs; one crossvein between R_5_ and M_1_, one between M_1_ and M_2_, one between M_2_ and M_3_ and one oblique crossvein between M_4_ and Cu_1_; Cu_1_ and Cu_2_ almost parallel with two crossveins between them; Cu_2_ bending sharply with an 90° angle at the level slightly proximad of the forking of M_3+4_; one crossvein between Cu_2_ and 1A (Figs 2E–H, 3B).

**Figure 4.**
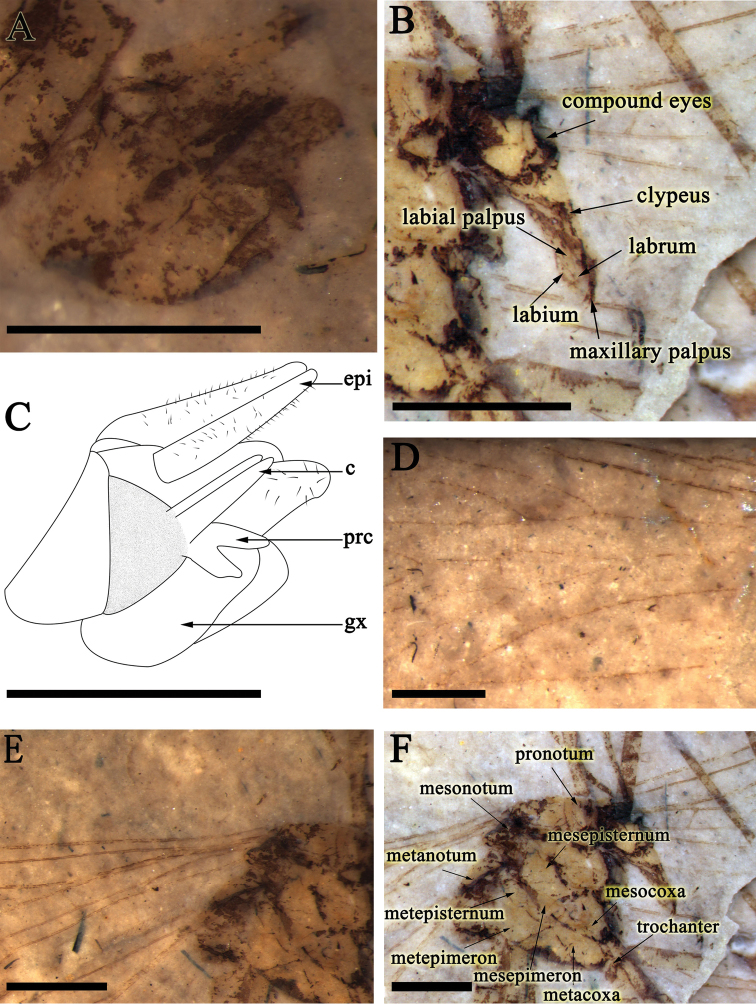
*Mongolbittacus speciosus* sp. n., holotype, photos under alcohol and a line drawing. **A** genitalia in lateral view **B** head **C** line drawing of genitalia in lateral view **D** vein M forking proximad of Rs forking in left forewing **E** anal field of left forewing; **F** thorax; Scale bars represent 0.5 mm in **A**, **C**, 1 mm in **B**, **D–F.** Abbreviations: c, cercus; epi, epiandrium; gx, gonocoxite; prc, procitiger.

Abdomen. Abdomen 6.5 mm long, with 9 visible segments. The ninth tergum (T9) connecting gonocoxite with dense short setae at the apex, epiandrium well-preserved with long setae on the surface; procitiger and cercus present in lateral view (Figs 1A–C, 4A, C).

##### Remarks.

*Mongolbittacus speciosus* sp. n. (Figs 1–4) is assigned to the genus *Mongolbittacus* based on the following generic diagnostic characters: R_4+5_ plus R_4_ distinctively curved; M_4_ simple; the ‘bittacid cross’ not aligned; wide posterior anal field; and the forking of M proximad of the Rs forking. *Mongolbittacus speciosus* sp. n. is distinguished from the other two species of *Mongolbittacus* by veins of 1A and 2A fusing at base, and 2A sharply curving to the posterior margin, as shown in the key below.

#### Mongolbittacus oligophlebius sp. n.

Taxon classificationAnimaliaMecopteraBittacidae

http://zoobank.org/D4B8FBAC-45F0-4A31-B805-108FB33AC7B1

Figs 5 , 6

##### Etymology.

The specific name *oligophlebius* denotes the wing venation is simple with only a few crossveins.

##### Holotype.

CNU-MEC-NN-2013009 P/C, part and counterpart. Forewing 12 mm long with a maximal width of 3.5 mm.

##### Paratype.

CNU-MEC-NN-2013014.

##### Horizon and locality.

Jiulongshan Formation, late Middle Jurassic, Daohugou Village, Ningcheng County, Inner Mongolia, China.

##### Diagnosis.

The posterior part of the “bittacid cross’ coinciding with the forking of M_3+4_; one oblique crossvein between R_2+3_ and R_4_ at the bifurcation of R_2+3_; and length of R_3_ 0.9 times as long as R_2+3_.

##### Description.

**Figure 5.**
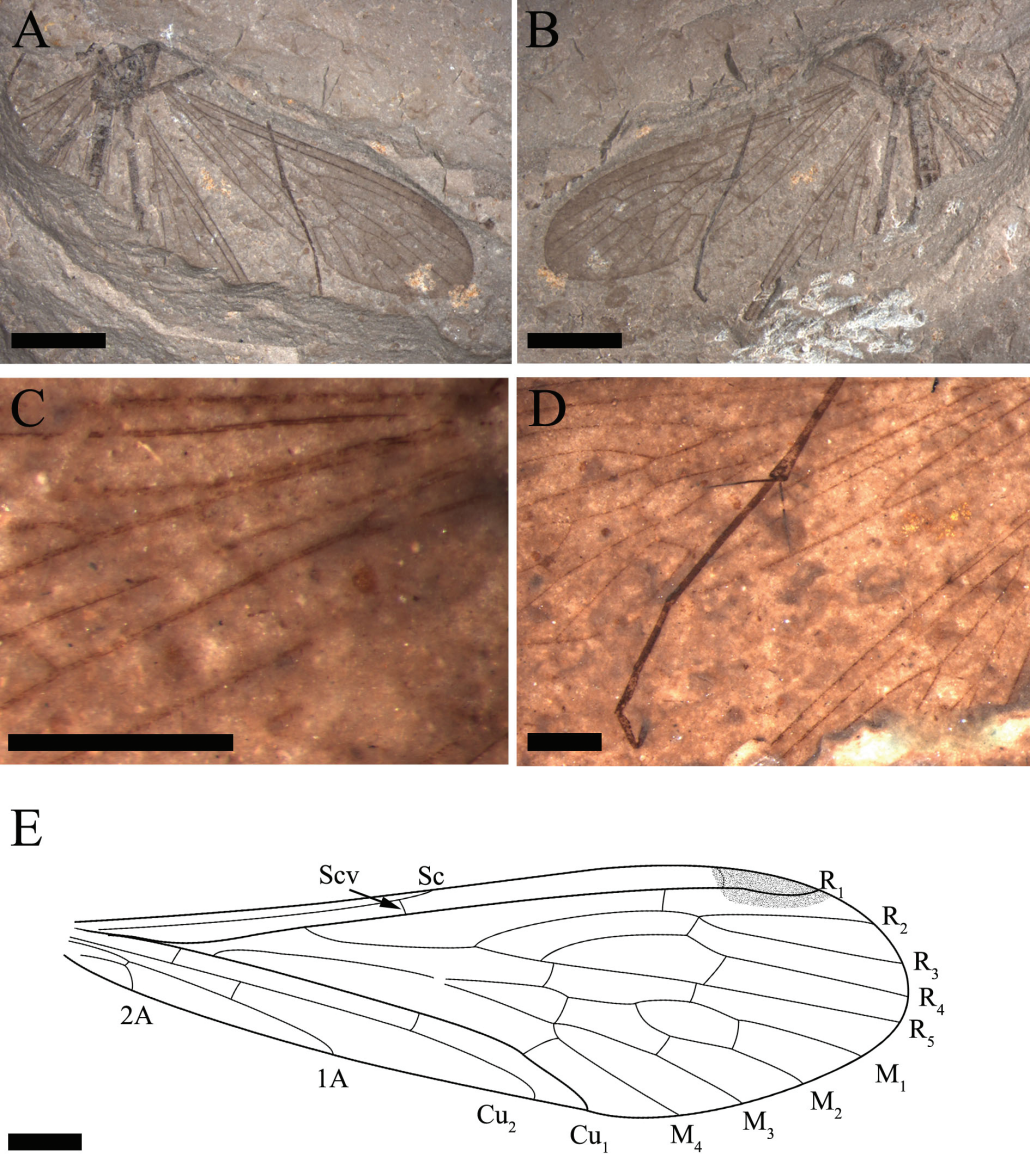
*Mongolbittacus oligophlebius* sp. n., holotype, CNU-MEC-NN-2013009 P/C. **A** photo of part **B** photo of counterpart **C** anal field of left forewing, under alcohol **D** photo of a leg, under alcohol **E** line drawing of right forewing of part. Scale bars represent 3 mm in **A–B**, 1 mm in **C–E.**

Poorly preserved with only one complete forewing and the basal part of one hind wing. But the mid-tibia with two long spurs and five tarsomeres well-preserved, covered by dense short setae (Fig. 5A, B, D).

**Figure 6.**
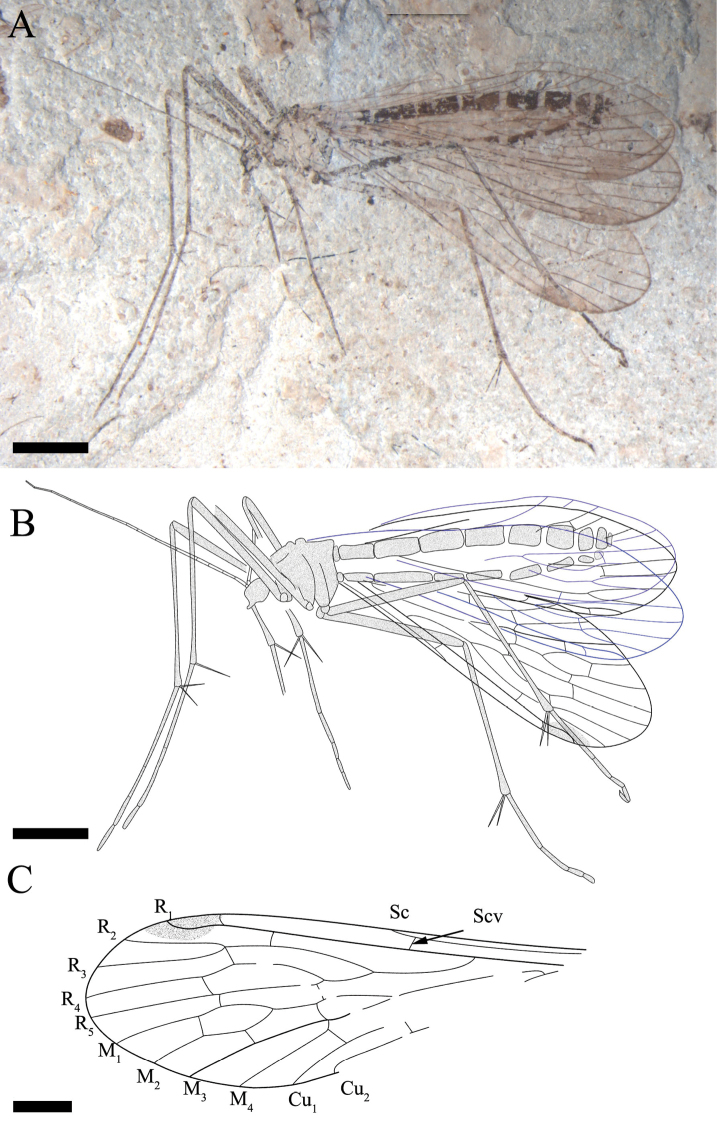
*Mongolbittacus oligophlebius* sp. n., paratype, CNU-MEC-NN-2013014. **A** photo **B** line drawing of part **C** line drawing of left forewing of part; Scale bars represent 2 mm in **A–B**, 1 mm in **C.**

Forewing. Sc reaching the anterior margin proximad of the forking of Rs, one crossvein between C and R_1_; one subcostal crossvein (Scv) between Sc and R_1_; Rs bifurcating into four branches, one crossvein between R_1_ and R_2+3_ and one oblique crossvein between R_2+3_ and R_4_; Rs arising from R_1_ at an acute angle; length of R_3_ 0.9 times as long as R_2+3_; one crossvein between R_4_ and R_5_; M with four branches and bifurcating proximad of the forking of Rs; the ‘bittacid cross’ not aligned; the posterior part of the ‘bittacid cross’ coinciding with the forking of M_3+4_; one crossvein between R_5_ and M_1_, one between M_1_ and M_2_ and one between M_2_ and M_3_; one crossvein between Cu_1_ and M_4_, Cu_1_ and Cu_2_ parallel with two crossveins between them; one crossvein between Cu_2_ and 1A; 1A reaching the posterior margin distad of the origination of Rs from R_1_; 2A bending sharply and reaching the posterior margin, a short crossvein between 1A and 2A (Figs 5C, E, 6C).

##### Remarks.

*Mongolbittacus oligophlebius* sp. n. (Figs 5, 6) is assigned to the genus *Mongolbittacus* based on the following generic diagnostic characters: R_4+5_ plus R_4_ distinctively curved; M_4_ simple; the ‘bittacid cross’ not aligned; posterior anal field broad; and 2A bending sharply and reaching the posterior margin. This new species is differentiated from *Mongolbittacus daohugoensis* and *Mongolbittacus speciosus* sp. n. by characters as shown in the key below.

#### Key to species of *Mongolbittacus* based on characters of the forewing

**Table:** 

1	Sc reaching the anterior margin proximad of the forking of Rs (Figs 3A, 5E)	**2**
–	Sc reaching the anterior margin distad of the forking of Rs	***Mongolbittacus daohugoensis* Petrulevičius, Huang & Ren, 2007**
2	1A and 2A fusing at base (Fig. 3A)	***Mongolbittacus speciosus* sp. n.**
–	A short crossvein between 1A and 2A (Fig. 5E)	***Mongolbittacus oligophlebius* sp. n.**

#### Exilibittacus

Taxon classificationAnimaliaMecopteraBittacidae

Yang, Ren & Shih, 2012

##### Type species.

*Exilibittacus lii* Yang, Ren & Shih, 2012.

##### Included species.

Type species, *Exilibittacus foliaceus* sp. n., and *Exilibittacus plagioneurus* sp. n.

##### Emended diagnosis.

Forewing: Sc reaching the anterior margin at the same level or proximad of the forking of R_4+5_; the ‘bittacid cross’ aligned, the posterior of the ‘bittacid cross’ distad of the bifurcation of M_3+4_; 1A terminating at the posterior margin at the same level or distad of the origination of Rs from R_1_. Hind wing: Rs with three or four branches, M with three branches and 2A absent.

#### Exilibittacus foliaceus sp. n.

Taxon classificationAnimaliaMecopteraBittacidae

http://zoobank.org/98FF3AC2-E493-45B7-B351-27B42685200A

Fig. 7

##### Etymology.

The Latin word of “*foliaceus*” means folliform, referring to the shape of the wings like leaves.

##### Holotype.

Female, CNU-MEC-NN2013010, in dorsal view. Body length 12.9 mm, forewing 11.7 mm long and 2.9 mm wide; hind wing 9.7 mm long and 2.6 mm wide.

##### Horizon and locality.

Jiulongshan Formation, late Middle Jurassic, Daohugou Village, Shantou Township, Ningcheng County, Inner Mongolia, China.

##### Diagnosis.

Forewing: pterostigmal crossveins (Pcv) absent, but 2A present. Hind wing: Rs with four branches and the bifurcation of Rs at the same level of the bifurcation of M.

##### Description.

**Figure 7.**
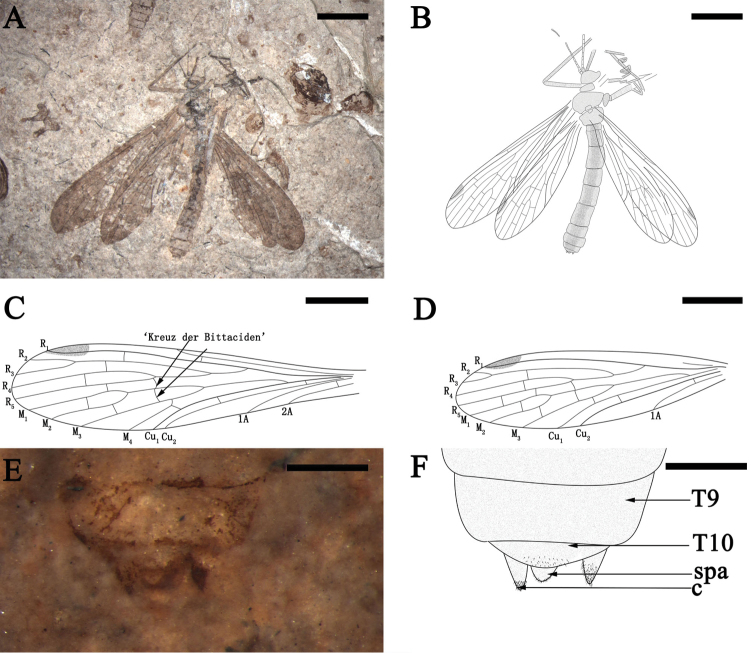
*Exilibittacus foliaceus* sp. n., holotype, CNU-MEC-NN2013010. **A** photo **B** line drawing **C** line drawing of left forewing **D** line drawing of left hind wing **E** photo of genitalia in dorsal view, under alcohol **F** line drawing of genitalia in dorsal view. Scale bars represent 3 mm in **A**, **B**, 2 mm in **C**, **D**, 0.5 mm in **E**, **F.** Abbreviations: T9, the ninth tergum; T10, the tenth tergum; c, cercus; spa, supraanale.

A female holotype preserved in dorsal view. Antenna filiform, scape, pedicel and part of other antennomeres preserved. The vertex of the head raised. Legs not well-preserved, covered with short setae; the fifth tarsomere folded against the fourth, a claw present (Fig. 7A, B).

Forewing. The base of wings narrow, pterostigma slightly dark. Sc terminating at the anterior margin proximad of the R_4+5_ forking; one subcostal crossvein (Scv) between Sc and R_1_; R_1_ running straight through pterostigma, without sagging; one crossvein between R_1_ and R_2+3_; Rs with four branches, R_4_ slightly curved at beginning and then parallel with R_5_; one crossvein between R_2+3_ and R_4_ and one crossvein between R_4_ and R_5_; M with four branches, M_3+4_ forking far proximad of the bifurcation of M_1+2_; the ‘bittacid cross’ aligned and gently curved, posterior part of the ‘bittacid cross’ reaching M_3_ distad of the M_3+4_ forking point; one crossvein between R_5_ and M_1_, one between M_1_ and M_2_ and one between M_2_ and M_3_; Cu_1_ and Cu_2_ almost parallel with two crossveins between them, one crossvein between M_4_ and Cu_1_; 1A and 2A simple and one crossvein between them; 1A reaching the posterior margin at the same level of the origination of Rs from R_1_; 2A reaching the posterior margin at the same level of the origination point of M (Fig. 7C).

Hind wing. With the same shape as the forewing. R_1_ running smoothly through pterostigma; pterostigmal crossveins (Pcv) absent; Rs with four branches; one crossvein between R_2+3_ and R_4_ and one between R_4_ and R_5_; the ‘bittacid cross’ aligned; M divided into three branches; two crossveins between R_5_ and M_1_, one between M_1_ and M_2_, one between M_2_ and M_3_ and one between M_3_ and Cu_1_; Cu_1_ and Cu_2_ parallel and with one crossvein between them. Vein 1A reaching the posterior margin at the level slightly proximad of the Rs originating from R_1_, one crossvein between Cu_2_ and 1A (Fig. 7D).

Abdomen. Abdomen 9.1 mm long, with ten visible segments. Female genital structure well-preserved from the dorsal view. Supraanale and cercus covered with small and short setae (Fig. 7A, B, E, F).

##### Remarks.

*Exilibittacus foliaceus* sp. n. (Fig. 7) is assigned to the genus *Exilibittacus* Yang, Ren & Shih, 2012 based on the following generic diagnostic characters: in forewing, Sc reaching the anterior margin proximad of the forking of R_4+5_ and the ‘bittacid cross’ aligned; and in hind wing, Rs with four branches while M with three branches. *Exilibittacus foliaceus* sp. n. is distinguished from the other two species as shown by the key below.

#### Exilibittacus plagioneurus sp. n.

Taxon classificationAnimaliaMecopteraBittacidae

http://zoobank.org/CCEF6E6E-B54D-43CB-8E24-F289597FB4C1

Figs 8 , 9

##### Etymology.

From Greek “*plagios*” (oblique) and “*neuron*” (vein), referring to oblique crossveins of the wings.

##### Holotype.

Female, CNU-MEC-NN2013013 P/C, in dorsal view. Abdomen length 8.3 mm, forewing length 9.3 mm with a maximal width of 2.3 mm; hind wing length 8.4 mm with a maximal width of 2.2 mm.

##### Horizon and locality.

Jiulongshan Formation, late Middle Jurassic, Daohugou Village, Shantou Township, Ningcheng County, Inner Mongolia, China.

##### Diagnosis.

Forewing Sc terminating at the anterior margin at the same level of the R_4+5_ forking; Vein 1A terminating at the posterior margin distad of the origination of Rs from R_1_.

##### Description.

**Figure 8.**
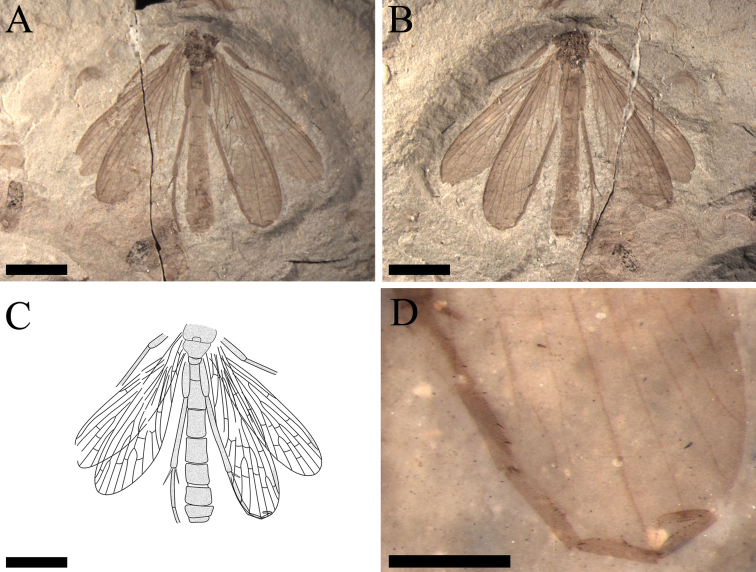
*Exilibittacus plagioneurus* sp. n., holotype, CNU-MEC-NN2013013 P/C, dorsal view. **A** photo of part **B** photo of counterpart **C** line drawing of part **D** photo of a hind leg, under alcohol. Scale bars represent 3 mm in **A–C**, 1 mm in **D.**

Female, small-sized, head not preserved but mesothorax and metathorax preserved. Legs partially preserved, one hind leg with five tarsomeres present but the pretarsal claw not preserved, the fifth tarsomere folded against the fourth; the first and second tarsomeres with several spines. (Fig. 8A–D)

**Figure 9.**
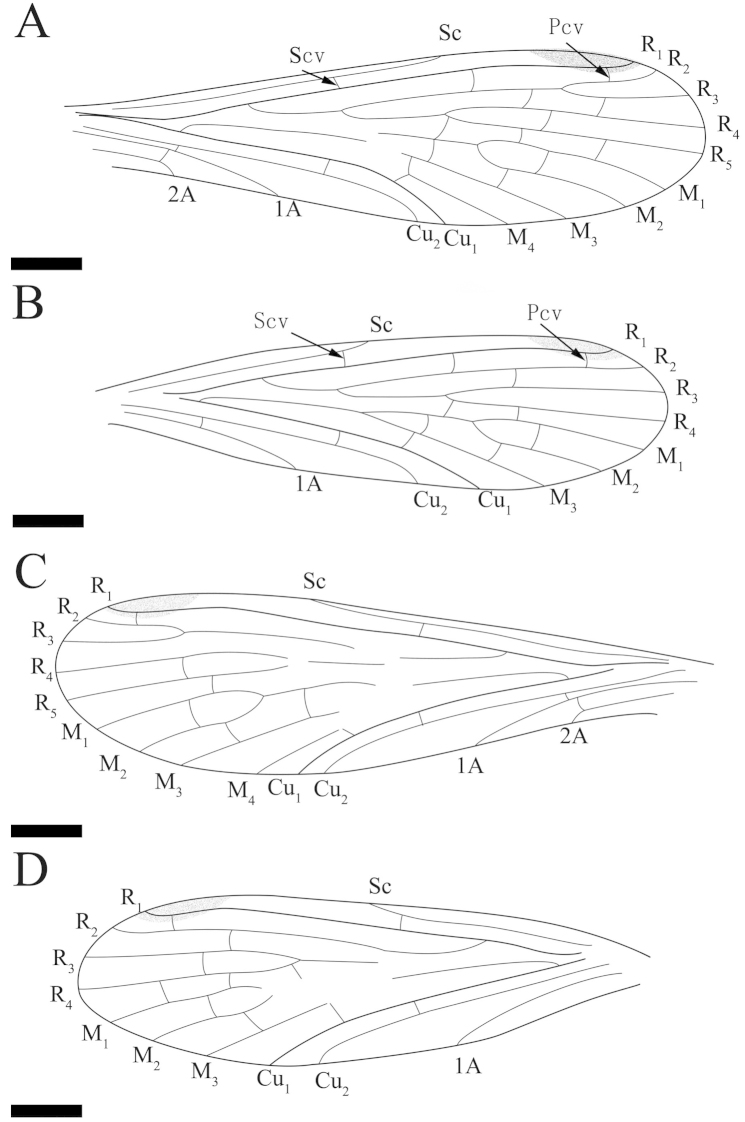
*Exilibittacus plagioneurus* sp. n., holotype. Line drawings of part. **A** right forewing of part **B** right hind wing of part **C** left forewing of part **D** left hind wing of part. Scale bars represent 1 mm in **A–D.**

Forewing. Wing narrow basally with obviously dark pterostigma. Sc long, reaching the anterior margin at the same level of the R_4+5_ forking; R_1_ not forking, one subcostal crossvein (Scv) between Sc and R_1_, Scv about 1/6 as long as the Sc length between Scv and the apex of Sc; one pterostigmal crossvein (Pcv) and one crossvein between R_1_ and R_2+3_; Rs with four branches, one crossvein between R_3_ and R_4_, one between R_2+3_ and R_4_ and one between R_4_ and R_5_; M with four branches, M_4_ base bending sharply; the ‘bittacid cross’ aligned, the posterior part of the ‘bittacid cross’ reaching M_3_ distad of the M_3+4_ forking point; two crossveins between R_5_ and M_1_, one between M_1_ and M_2_ and one between M_2_ and M_3_; Cu_1_ ending before the forking of R_4+5_, one crossvein between M_4_ and Cu_1_, one between Cu_1_ and Cu_2_; one short crossvein between Cu_2_ and 1A; 1A terminating at the posterior margin distad of the origination of Rs; 2A ending proximad of the originations of Rs and M, one crossvein between 1A and 2A (Fig. 9A, C).

Hind wing. Sc short, reaching the anterior margin before the forking of R_4+5_, one crossvein (Scv) between Sc and R_1_; R_1_ smooth and not sagging through the pterostigmal area; one pterostigmal crossvein (Pcv) present. Rs with three branches; one crossvein between R_1_ and R_2_, one between R_2_ and R_3_ and one between R_3_ and R_4_; M with three branches; two crossveins between R_4_ and M_1_, one between M_1_ and M_2_ and one between M_2_ and M_3_; the ‘bittacid cross’ not aligned; one between M_3_ and Cu_1_ and one between Cu_1_ and Cu_2_. Vein 1A terminating at the posterior margin distad of the origination of Rs, one crossvein between Cu_2_ and 1A (Fig. 9B, D).

Abdomen. Ten segments visible, genital segments not preserved (Fig. 8A–C).

##### Remarks.

*Exilibittacus plagioneurus* sp. n. (Figs 8, 9) is assigned to *Exilibittacus* Yang, Ren & Shih, 2012 based on the following generic diagnostic characters: in forewing, Sc reaching the anterior margin at the same level of the forking of R_4+5_ and the ‘bittacid cross’ aligned, and in hind wing, M with three branches. The new species is differentiated from *Exilibittacus lii* and *Exilibittacus foliaceus* sp. n. by characters shown in the key below.

#### Key to species of *Exilibittacus* based on characters of both fore- and hind-wings

**Table:** 

1	Rs with four branches in hind wing (Fig. 7D)	***Exilibittacus foliaceus* sp. n.**
–	Rs with three branches in hind wing	**2**
2	1A terminating at the posterior margin of the forewing distad of the origination of Rs (Fig. 9A)	***Exilibittacus plagioneurus* sp. n.**
–	1A terminating at the posterior margin of the forewing proximad of the origination of R	***Exilibittacus lii* Yang, Ren & Shih, 2012**

## Supplementary Material

XML Treatment for Mongolbittacus

XML Treatment for Mongolbittacus speciosus

XML Treatment for Mongolbittacus oligophlebius

XML Treatment for Exilibittacus

XML Treatment for Exilibittacus foliaceus

XML Treatment for Exilibittacus plagioneurus
